# Hospital and Institutional News

**Published:** 1911-12-09

**Authors:** 


### HOSPITAL AND INSTITUTIONAL NEWS.

### WORKERS' PROTESTS ON BEHALF OF HOSPITALS.

We are glad to learn that our advice is being followed by the organisation of a campaign in the industrial centres of England to protest against the position in which the voluntary hospitals are likely to be placed under the Insurance Bill. We hope that, as has been urged repeatedly in these columns, these workers' protest meetings will serve the double purpose of saving not only the voluntary hospitals, but the working classes also from the absence of hospital treatment with which the Bill threatens them at present. The interests of the working classes have been always so much bound up with those of the hospitals that the present is a unique opportunity for each to stand by and to uphold the interests of the other. Every hospital secretary should second the campaign where it has been started, and initiate it where it has not yet begun.

### MR. GEORGE AND MUNICIPAL HOSPITALS.

The day after the great meeting of the British Hospitals Association had been reported in the papers, a long letter appeared criticising Mr. Lloyd George's proposed Insurance Bill in its baneful effects upon the voluntary hospitals in the light of a recent pamphlet by a medical man entitled "A National Medical Service." Mr. Lawson Dodd has written this at the behest of the Fabian Society, which publishes it as a tract, and in it he goes so far as to say "the maintenance of all hospitals out of imperial and local funds, and their management by the community, will be the first step towards educational efficiency in the profession." Begging the question whether it is not absurd to identify Mr. George with the Fabian Society, most medical men will feel that the nationalisation of the hospitals, by converting the then paid medical staff probably into whole-timers, may tend to lower medical standards, for the prestige of a staff appointment and its indirect emoluments would cease to lure men who are no longer permitted to convert them into the successes of private practice.

### THE MEDICAL MEMBER OF THE INSURANCE COMMISSION.

On Monday afternoon the Chancellor of the Exchequer announced in the House of Commons, in reply to a question, that the Government had appointed Mr. James Smith Whitaker as the medical member and Deputy Chairman of the Insurance Commission. Mr. Whitaker qualified M.R.C.S. and L.R.C.P. in 1891, and is the Medical Secretary of the British Medical Association. What his qualifications are for dealing with so important a work as organising the system of National Insurance under the Bill we are, we confess, quite at a loss to know, but probably Mr. Lloyd George has decided upon this appointment in consultation with his expert advisers who have already given him such useful advice with regard to the medical matters in the Bill. It is to be hoped, however, that the public may be taken into confidence, and given the reasons for this particular choice. The post of direct representative of the medical profession on the Commission is a most important and responsible one, and, we venture to think, it should be entrusted to one who has had some experience of insurance work, and who has studied the working of the Government system in Germany and Switzerland. We do not know if Mr. Whitaker has that experience, but he has not identified himself prominently with insurance work, and we doubt whether the profession will cordially approve of the choice that has been made.

### THE IMPRESSION CREATED BY THIS APPOINTMENT

We express this opinion without the least desire to deprecate the claims which Mr. Whitaker may possess to be regarded as a suitable representative, but it seems to us that in view of the action which the Association has taken in regard to the Bill, it would have been better had Mr. Lloyd George chosen some medical man outside its ranks, and one who is generally known to the profession. The impression naturally will be that the Chancellor has attempted to buy the goodwill of the Association by making this appointment. If that is really the case, he may be assured that the imbroglio created by his Bill will not be bettered, but rather made worse, by it.

### "THE LATEST THING IN THEATRE-UNITS."

In remarking last week that the *x*-ray room in the new theatre-unit at the North Riding Infirmary is entered from the anæsthetic-room, our quotation from Mr. Postgate's pamphlet should read "no inconvenience arises from this fact, as work in the two rooms is not carried on simultaneously."

### A VICAR ATTACKS THE SUNDAY FUND.

Parish magazines have seldom excited so much feeling as the latest issue of St. Andrew's parish magazine, Newcastle, has aroused. In it the Vicar, Canon Moore Lister, says that he is sending his congregation's collection direct to the medical charities on the ground that "the expenses of the Newcastle Sunday Fund (as published) are more than 12 per cent. of the whole income. The expenditure includes £120 for clerk's salary and about £60 for office rent and expenses." Canon Lister proceeds: "Fourteen pages of the annual report are filled with the speeches of seven gentlemen (mostly verbatim and in very large type) at the annual meeting on September 15, when (including these speakers) there was an attendance of only ten. These expenses must be reduced before we can again send our contributions through the fund." Then, as illustrating the position of his congregation, he says, "The sum of our last two collections was above £97, of which the whole goes to the medical charities. If we had sent that money through the fund those charities would only have received £85." Canon Lister concludes by hoping that "with the appointment of a new honorary secretary to the fund there will be great economies in its working expenses, and great tact in claiming and obtaining representation for the contributors to the fund in proportion to their contributions upon the governing bodies of the charities they support." He finally sums up his case by saying "There is no question but that for some years past the fund has come to a standstill in the rut of routine." What answer have the secretary and the committee of the Newcastle Sunday Fund to make to this trenchant indictment?

### WIGAN'S NEW MEDICAL OFFICER.

No fewer than forty-two candidates applied for the post of Medical Officer of Health for the county borough of Wigan. The successful applicant was Mr. F. E. Wynne, formerly Medical Officer of Health for Leigh, who succeeds to Mr. J. R. Hutchinson, newly become a medical inspector under the Local Government Board. Mr. F. E. Wynne graduated at Dublin 1894, and has been honorary consulting surgeon to the Leigh Infirmary. Mr. Wynne is also an author, having published a work entitled "Fortune's Fool."

### AN INTER-STAFF SQUABBLE AT SWANSEA.

In moving on Tuesday that "of the executive representatives of the honorary medical staff on the Board of Management four shall be the senior members of the honorary consulting staff" of Swansea Hospital, Dr. Jabez Thomas urged that himself and two other members of the senior consulting staff ought to be members of the Board. This provoked Dr. Griffiths to say that the doctors should be elected by the medical staff, at which Dr. Thomas protested vigorously. The discussion continued with some heat till finally stigmatised as a "doctors' squabble," but, just as the atmosphere had apparently cleared, Dr. Rawlings, one of the two medical men mentioned by Dr. Thomas, jumped to his feet and suddenly withdrew his candidature. To make the topsy-turvydom of this extraordinary meeting complete, those present decided, in spite of Dr. Rawlings' emphatic refusal, to put his name forward for the post. Mr. Aeron Thomas presided.

### A HOSPITAL WITHOUT AN ARCHITECT.

On Monday last the new Cottage Hospital at Horden, near Darlington, was opened by Mr. J. J. Prest, J.P., of Hardwick Hall, agent to the local collieries. Mr. Hedley Mason presided, and reminded the large gathering that the idea of the new hospital had been introduced by Dr. Muir, and was the outcome of a co-operation between the colliers and their employers. Not the least interesting fact about the building is that no architect has been appointed for the work; we are therefore sorry that the name of the designer or surveyor or the responsible drafter of its plans was not mentioned. The institution would be, said Dr. Morison, a half-way house for casualties, and perhaps will prove the forerunner of a larger hospital.

### QEEEN ALEXANDRA AND HOSPITAL SECRETARIES.

Some months ago we published a preliminary list of those memorials to King Edward which, as was so often the case, took the form of hospital extension and improvement all over the country. We noted then how powerful his late Majesty's name had proved in getting things done which hitherto had been delayed, and how much hospital progress could be dated from the country's desire to commemorate the year of his death. It is now stated that by Queen Alexandra's command, Colonel Sir James Gildea of 11 Hogarth Road, S.W. "has undertaken to collect particulars, for publication in due course, of all memorials, of whatever description . . . to his late Majesty." Hospitals having so frequently been made the object of these memorial schemes, we invite all hospital secretaries whose institutions have been connected with them to communicate with Colonel Sir J. Gildea "as the memorials are completed, when he will be glad to send particulars of the information required." We feel sure that the hospitals will be only too glad to assist in this way in carrying out Queen Alexandra's express wishes.

### SUNDAY CINEMATOGRAPHS.

In our issue of September 30, page 679, we dealt with the action of the City of London Guardians in regard to their annual donation of £21 to the London Hospital. This action was based upon objections to the course adopted by the London Hospital authorities, which, be it remembered, they had a perfect right to pursue, in taking money from Sunday cinematograph exhibitions in conjunction with two or three other Metropolitan hospitals. The annual meeting of the constituents of the Hospital Sunday Fund is to be held this month, and, in addition to the falling off in the congregational collections in London during 1911, further evidence has been before the Council of the growing opposition to Sunday cinematograph shows and its probable effects upon the Hospital Sunday Fund. In these circumstances the Council, we understand, approached the Committee of the London Hospital, and after fully going into every side of the question the London Hospital authorities have decided to relinquish the present income, which is a substantial one, which they derive from Sunday cinematograph shows, and to discontinue the London Hospital's connection with these exhibitions. This wise and prudent decision does honour to the spirit, judgment, and wisdom of the London Hospital authorities, and will be welcomed by the public and hospital opinion. We hope that this action, taken by the London Hospital with the object of preventing any injury to the Hospital Sunday Fund from the holding of cinematograph exhibitions on Sunday, will be as widely recognised as it is undoubtedly appreciated. Then, as is but just, instead of losing income by the generous course they have taken, the London Hospital authorities should promptly receive an even larger addition to their voluntary revenue than any sum they may lose by discontinuing Sunday cinematograph shows.

### SUNDAY CINEMATOGRAPHS: WAKE UP, PARSONS!

We are convinced that under proper regulations, so far from being an evil, Sunday cinematograph shows, in this country, might be made of immense value and do momentous good to the people. Why do not the clergy and ministers of religion rise to the occasion, undertake the management of Sunday cinematograph shows themselves, select interesting and instructive subjects for illustration by pictures, and organise a band of competent men and women to give explanatory addresses whilst the cinematograph show is proceeding? When the illustrations appeared on the screen an earnest and clever speaker could readily secure that each audience would be not only amused but benefited intellectually and morally by attending these Sunday shows. We are confident that this field, when wisely cultivated, free from cant and unctuousness, can be made to yield to the churches abundant fruit. For the people it would bring benefits fruitful in good and full of pleasant entertainment and instruction.

### SANDOW'S MEDICAL EMPLOYEES.

At the recent session of the General Medical Council the cases of two practitioners who were charged at the previous session, six months ago, with "infamous conduct" on account of their association with the "Sandow Institute" were again investigated. On the previous occasion these two doctors were warned that the allegations against them were proved to the satisfaction of the Council, and a definite decision was deferred in order to give them an opportunity of considering their position and amending their conduct. On November 28, when these cases came up again for review, one of the defendants declared that since May he has had no connection with the Sandow Institute; and the Council accordingly decided not to subject him to the penalty of erasure from the Register. The other practitioner, it appears, is still recalcitrant, and has resumed his connection with the "Institute"; the case on his behalf was argued by Lord Robert Cecil, and the chief point relied on is that the Sandow Institute has now ceased to advertise for the purpose of procuring patients. This announcement, however, is quickly contradicted by a renewal of its practice, and its exaggerated claims in respect to the cure of diseases are again to be circulated broadcast. On the legal points raised by Lord Robert Cecil the Council seems to have been a little uncertain, and eventually it was decided to give the defendant another six months in which to repent. This postponement is a weak and unsatisfactory way round the difficulty, and it were to be wished that the Council had acted with the courage of its convictions—if it has any. The result of this vacillation has been to stiffen the backbone of the accused practitioner, who now publicly announces his intention to continue in his course and to defy the Council, which will thus have no option but to strike him off or else swallow its own words.

### THE FIRST USE OF X-RAYS IN ENGLAND.

Dr. Garson took the chair at a meeting in connection with the Port Sunlight Literary and Scientific Society, at which Dr. Thurston Holland, of Liverpool Royal Infirmary, and an *x*-ray pioneer in this country, gave an interesting account of the history of the invention and its present field in surgery. It was his very good fortune to be one of the first men in England to use *x*-rays in a practical way. His friend Robert Jones was quick to recognise the possibilities of the invention. He consulted Professor Oliver Lodge, then connected with their University, as to the locating of a bullet supposed to be in the hand of one of his patients. They rigged up an apparatus, and after repeated trials obtained a plate showing an undefined but convincing shadow of the bullet just before the joint of his wrist. The fame of the discovery soon spread. Liverpool was not backward in the matter, and the field had been constantly enlarged in all directions. It became necessary that a hospital should have a department for *x*-rays equipment, and a man specially competent to use it. As a first instance of the use of *x*-rays in this country Dr. Thurston Holland's account is worthy of preservation.

### AT SCHOOL ON A BANDSTAND.

The St. Marylebone dispensary for the prevention of consumption has adopted the original plan of holding open-air classes for phthisically predisposed children on one of the bandstands in Regent's Park. The school which is under the control of a medical officer, Dr. Sutherland, was started by private enterprise with the assistance of a grant from the C.O.S., but the past three months have proved it to be so successful that the dispensary is now wholly responsible for its welfare. The number of children who attend is about thirty, and the school is open from nine to five, with a short break at eleven and a two-hours' interval at twelve, when dinner and organised games are enjoyed. The tuberculosis dispensaries from the first are showing commendable originality in prosecuting their work, of which the present example is an excellent type.

### A PATIENT EXPELLED AT MANCHESTER.

The expulsion of an inmate of the Northern Counties Hospital for Incurables at Mauldreth near Manchester was made the subject of some discussion at a meeting held last week. The Rev. W. H. Smartt alleged that the man in question, who, he said, was married and in very poor circumstances, had been expelled because he "had refused at the dictation of the secretary, Mr. William Ferguson, to attend certain religious services." The chairman, Mr. Alfred Crewdson, in reply said that there were sometimes patients who were not inclined to observe the rules of the hospital loyally and that the board were entitled to remove such patients under the rules. The man alluded to was one of this class, and it was unnecessary for the board to formulate specific charges. The board decided that the man alluded to was an undesirable inmate. We can hardly believe that Mr. Smartt's fears of religious intolerance had any foundation, and it is only reasonable to allow the governing bodies of voluntary institutions to decide who shall and who shall not remain under their care. If discharge were to be taken as a stigma, no one would be allowed to give his servants a month's notice. If the payment of wages gives the employer liberty of choice, the bestowal of benefits should no less do so. That it must, in practice, Mr. Smartt has failed to understand.

### A CURIOUS ROYAL GIFT.

The West London Hospital at Hammersmith is reported to have received one of the oddest gifts to hospitals of recent times—namely, two models of an elephant and a horse, with elaborately embroidered caparisons. It is said that the latter were worked by a girl of fourteen, and that they belonged originally to a collection of Indian curios which had been given to the Queen, and which she is now distributing among the hospitals. It would be interesting to know how the hospital proposes to make use of this original present.

### SOUTHAMPTON'S LONGEST SUBSCRIBER.

An interesting example of the way in which the oldest subscriber to the Southampton and South Hants Hospital regarded that institution is found in the will of the late Mr. Edmund Chipperfield, a retired chemist, who has just died at the age of ninety-three, leaving an estate of the gross value of some £80,000. In his will he wrote: "I am reminded that I am the oldest and longest subscriber to the Southampton and South Hants Hospital, and therefore that it becomes me, before I go home, to will something towards its continuance and support. I therefore will an annuity of £200, to be paid half-yearly." Mr. Chipperfield is the old type of hospital subscriber which the trend of modern legislation is threatening to remove. We hope his name will be honoured by his hospital in some permanent fashion to remind the future of his constant interest in hospital work. The secretary, Mr. Hall, should see that this is not forgotten.

### THE MAKING OF MODERN SUPERINTENDENTS.

In giving an account last week of the City of London Hospital's festival dinner, reference was made to the services of the secretary, Mr. George Watts, and to those of the resident medical officer, Mr. W. W. Jameson, M.A., M.B. Mr. Watts was appointed secretary of the City of London Hospital for Diseases of the Chest in 1910, having been engaged in hospital work for thirteen years. He began his business career in the City, and from there passed to the Brompton Hospital, where he acted as clerk in the secretary's office from 1897 to 1903. In May of that year Mr. Watts was appointed secretary to the Hampstead General Hospital, which under his direction much extended its work by new buildings (erected at a cost of £60,000, of which £45,000 was raised during his tenure of office), which increased the accommodation from 35 to 114 beds. At that time, too, the scheme of amalgamation with the North-West London Hospital was taking effect, and a new out-patient department with emergency beds was being built in Kentish Town. While secretary at Hampstead General Hospital Mr. Watts had the organisation of three festival dinners, his experience in the arranging of which was evident by the success of the City of London Hospital's dinner last week. When Dr. T. Glover Lyon replied for the medical staff he referred to the services that Mr. W. W. Jameson, the resident medical officer, had rendered to the organisation among patients and staff at the Victoria Park Hospital. Mr. Jameson became resident a year ago, having previously served as junior and then senior house surgeon to the Prince of Wales's Hospital, Tottenham, but from May to October 1910 he had acted as house physician at Victoria Park, and, in the absence of the then R.M.O., as medical officer. Mr. Jameson is a graduate in arts and medicine of Aberdeen University, and his record is interesting in the light of his success as superintendent.

### MR. CHURCHILL AND BANSTEAD MENTAL HOSPITAL.

Lord Middleton is to be congratulated upon his speech at the London County Council on November 28, when the Council had under consideration a report from its Asylums Committee dealing with the release and subsequent re-arrest of a criminal lunatic from Banstead Mental Hospital. Widespread publicity was given at the time to a statement made in the police court that the order for the man's release was made by the then Home Secretary against the wishes of Mr. Percy Spark, the medical superintendent of Banstead Mental Hospital. As a matter of fact the committee's report clearly showed that the medical superintendent himself suggested to the Home Office that the man might be safely released. Lord Middleton, in view of this, took the opportunity to express his regret for a speech which he delivered at the time criticising Mr. Churchill's action. The matter thus ended satisfactorily, but it should serve to check those who fancy that the authority of a medical superintendent can lightly be set aside in a matter that affects his patients. If that were so, institutional administration would become impossible.

### MENTAL HOSPITALS AND COMING LEGISLATION.

The General Purposes Committee of the London County Council have just issued a report of an important deputation which recently waited upon the Home Secretary from that body on the question of the care and control of the feeble-minded. The deputation included the chairman of the Asylums Committee, Mr. A. O. Goodrich, in view of the importance of the matter in relation to the mental hospitals service. Mr. McKenna, in his reply to the deputation, stated that he had in draft an Inebriates Bill and also the heads of a Bill for dealing with the feeble-minded, both of which he trusted would become law next session. The Home Office agreed with the Council's contention for the establishment of a central authority for the care and control of the mentally defective. As regards the treatment of inebriates, he foreshadowed legislation whereby magistrates would in every case be enabled to commit an habitual inebriate to receive reformatory treatment, the expense of which might be assisted from the Treasury. The President of the Board of Education, Mr. J. A. Pease, M.P., also replied to the deputation, and referred to the necessity for making compulsory the operation of the Elementary Educative (Defective and Epileptic Children) Act, 1899, thus reducing the number of adult cases. Mental hospital officers will await with interest this proposed legislation, and we shall be curious to see what methods will be suggested for the relief of their burdens in dealing with lunacy.

### THE FOUNDER OF HOSPITAL SUNDAY IN BRISTOL.

The recent resignation of Mr. E. J. Swann from the treasurership of the Lord Mayor's Hospital Sunday Fund in Bristol has been much regretted locally. The Fund has been in existence for fifteen years, and for fourteen of them Mr. Swann has been its treasurer, and though ably assisted by Mr. Mortimer Harley, the honorary secretary, and by Mr. J. H. Reed, on him has fallen in the past the main responsibility for the Fund's success. This success has been not only financial, for nearly twenty years ago Mr. Swann and seven friends resolved to change the unsatisfactory system of collecting money which used to obtain in Bristol. Any rivalry there may be to-day between the Bristol medical charities is a friendly one, thanks largely to the inspiration of Mr. Swann and his friends. His successor in the treasurership is Mr. W. Garnett, who has not let the grass grow under his feet, having already, we believe, succeeded in establishing a new centre of collection.

### THE COMMITTEE AND ITS TASK AT BRIDGWATER

The new president of the Bridgwater Hospital is Mr. R. A. Sanders, who is the member of Parliament for the division. The honorary secretary is again Mr. E. Trevor, but the committee of management includes three new members, Mr. F. H. Gould, Mr. H. J. Masding, and Mr. W. Stilling. These, it is expected, will have a busy year in front of them, as owing to the increase in the housekeeping accounts, consequent upon an increase in the staff, to the present debt, and to the urgency with which the medical staff calls for the enlargement of the hospital, the necessity for new sources of income is increasing at the very time when the old sources hardly maintain their annual yield. A fund for building a new wing is to be started, and local interest is to be aroused by a carnival and other means. In short, it will be interesting next year to see how these officers under their chairman, Mr. R. Y. Foley, have tackled the difficulties of the present moment, and what success Mr. Trevor and Mr. Stilling can achieve.

### HOSPITAL AFFAIRS IN THE CHANNEL ISLANDS.

On November 28 the States met at Jersey to consider, among other matters on the agenda paper, the report of the special committee appointed to deal with the hospital question in the Islands. The Hôpital Général is, we believe, the only institution in Jersey for the relief of chronic invalids who cannot be nursed in their own homes, since the Dispensary will not admit such patients. It is at present a combination of poor-house and infirmary, but its work in the latter capacity has been steadily increasing in dimensions, necessitating a corresponding increase in expenses of upkeep. Thus the cost per inmate has risen from approximately £18 in 1899 to £29 this year. A sub-committee appointed in 1909 went very fully into the whole question, and reported that certain modifications should be made so as to allow for an increased medical staff, improved nursing, re-classification of inmates from a medical point of view, and the provision of better accommodation. The States decided that there was no need to increase the medical staff, and the present committee in its report strongly dissent from the view taken by previous committees that the hospital arrangements should be modernised. "The sick wards," the report states, are "infirmary wards where sick inmates can be efficiently cared for without having recourse to the ambitious and misleading title of hospital wards." This view is one which we suggest the good people of Jersey should strongly protest against. A modern infirmary ward differs in no respects from a hospital ward, and to consider that poor patients should have less modern treatment than patients in a voluntary hospital is to lose sight totally of the intention which has caused the establishment of such infirmary wards.

### A POLICY DETRIMENTAL TO PATIENTS.

The short-sighted policy of "setting our face steadily against the development of the institution on hospital lines in whole or in part," which is based on the fear that the expenditure must increase out of all proportion to the efficiency of the institution—for that is the only reason we can read in this extraordinary report—may amuse those of us who take a calm outsider's view of the matter, just as Mrs. Partington's vain attempt to scrub the Atlantic out of her cottage may have amused whoever was present on that memorable occasion. But it is a policy that will undoubtedly inflict serious detriment upon the unfortunate patients who are destined to be admitted to the Hôpital Général, and for that reason we hope that public opinion in Jersey will wake up and give the matter earnest and unprejudiced attention.

### THE NURSING ARRANGEMENTS AT THE JERSEY HOSPITAL.

The report, we notice, expresses the opinion that there should be no change in the present nursing arrangements. At present the male wards are in charge of so-called male nurses, who, we are informed, are untrained attendants. While it is advisable that such attendants should be employed in male wards, as they are in most hospitals under the Poor Law, we think that all authorities are with us in insisting that such wards should at the same time possess a staff of skilled and well-trained nurses who are duly qualified for work in a sick ward. There can be no excuse for omitting to supply this deficiency in Jersey. It is stated in the report that "there is much medical opinion adverse to this view" of replacing the unskilled male attendants by trained female nurses. We are surprised to hear such an opinion seriously voiced, for we had no idea that our medical *confrères* in the Channel Islands had retained the conservative ideas of the profession that were last upheld at the time of the Crimean War. We prefer to believe that their opinions have been erroneously recorded by the committee.

### WHAT THE MEDICAL STAFF SHOULD DEMAND.

At any rate, it is to be hoped that the medical officers of the institution will take the matter into their own hands and insist that they should be given a proper nursing staff modelled on the lines which obtain in other institutions of similar size and importance. It is amazing to contemplate a modern institution which still exists on an administration which appears to be based on that utter want of a realisation of what are the indispensable essentials of institutional treatment that characterised our infirmaries when Howard reported upon them. Here again we commit the case to the court of common sense and public opinion in Jersey, in the firm assurance that, once the absurdity of tinkering with a subject that demands thorough reorganisation is realised, it will not be long before the much-needed improvements are effected.

### FIFTY YEARS OF PUBLIC HEALTH.

Mr. Benjamin Kemp, having just attained the age of seventy-seven, has sent in his resignation as Medical Officer of Health for Horbury, near Wakefield, Yorkshire, a post which he has held for fifty years. Mr. W. Moorhouse, J.P., the chairman of the Wakefield Guardians, who accepted the resignation, said that so long a tenure of office was unprecedented in the history of this Union. It is also worth noting that the whole of Mr. Kemp's professional life has been spent in the public health service. He has never apparently, like his colleagues, tried private practice before adopting the *rôle* of officer of health. In 1859 Mr. Kemp qualified M.R.C.S., in 1861 he became L.S.A., and in that year, the first in which he completed his medical examinations, he was appointed M.O.H. for Horbury. It is an interesting record, and might one day become a classic example of the undisturbed advantages which the public health service offers to medical men. During this half-century, it is stated, the population of the district has doubled, which has led to the doubling of the emoluments. To-day they are £34—in 1861 they were £16 only, plus the value of the fees belonging to the public vaccinator, which is held jointly with the other office.

### "A WORKING-MAN'S HOSPITAL"

In these words Mr. Andrew Motion described the City of London Hospital for Diseases of the Chest at a dinner we reported last week for the special object of raising £3,000. That the institution is such, the speaker pointed out, was due to the support of working-men, who not only had raised practically £900 of this sum, but who were the chief rank of its supporters. He warned against the trend of legislation, which he feared would do harm to the hospitals, and asked working-men whether they thought they would be treated with the same courtesy as that which they received nowadays if the hospitals were nationalised. The sum raised is a striking proof of the affection of the working-men for this hospital, which we believe they would not forget to express emphatically, supposing the Insurance Bill were ever made, unamended, the subject of a referendum.

### THE MIDDLESEX HOSPITAL'S DECISION.

In spite of the success of the funds raised on behalf of the Middlesex Hospital by the late Prince Francis of Teck, and of the Memorial Fund raised to his memory, which now totals £28,240, the sale of certain freehold properties has been sanctioned. These properties lie mostly in Brixton and include a public-house, residential houses, and a contractor's yard, which are expected to realise nearly £18,000. Mr. Andrew Clark presided at the governor's court which sanctioned the sale in order to meet both extraordinary and current expenditure. In view of the great efforts that have been made during the past year and more, we hope that the increased public interest taken in the Middlesex Hospital will be steadily maintained.

### THIS WEEK'S DRUG MARKET.

There has been some improvement in business during the week, and it looks as if the year may end fairly well so far as the drug market is concerned. The main objects of interest continue to be opium, morphine, and codeine, the high values of which are well maintained and appear likely to increase still further. Interest in quinine has subsided to some extent, but a fair business continues to be done. Glycerine is unchanged in price, but any alteration would probably be in a downward direction; under the circumstances glycerine is not an article which it is advisable to buy in quantity at present. A fair business has been done in eucalyptus oil, and the price tendency is in an upward direction. Cod liver oil continues to decline in value. Oil of cloves is cheaper. Carbolic acid is again slightly dearer. Sugar of milk still tends downwards in price. Saffron is rather dearer. At the recent public sale of drugs in Mincing Lane higher prices were paid for cardamons and ipecacuanha, but Cape aloes and honey were slightly cheaper.

